# The Global Transcriptional Response of Isolated Human Islets of Langerhans to Glucagon-Like Peptide-1 Receptor Agonist Liraglutide

**DOI:** 10.5402/2012/608672

**Published:** 2012-09-29

**Authors:** Xiaoning Zhao, Yongming G. Tang, S. Vincent Wu, Charles Wang, Ricardo Perfetti, Nasif Khoury, Dehong Cai, Fang He, Xiaogang Su, Vay Liang W. Go, Hongxiang Hui

**Affiliations:** ^1^Center of Metabolic Diseases, Beijiao Hospital, Southern Medical University, North 1838 Guangzhou Road, Guangzhou 510515, China; ^2^International Center for Metabolic Diseases, Southern Medical University (SMU), 8 Floor, Life Science Build, North 1838 Guangzhou Road, Guangzhou 510515, China; ^3^Department of Medicine, Cedar-Sinai Medical Center, Los Angeles, CA 90048, USA; ^4^Department of Medicine, VA Greater Los Angeles Health Care System, Los Angeles, CA 90073, USA; ^5^UCLA Center for Excellence in Pancreatic Diseases, David Geffen School of Medicine at UCLA, Los Angeles, CA 90095, USA; ^6^School Biotechnology, Southern Medical University, North 1838 Guangzhou Road, Guangzhou 510515, China

## Abstract

GLP-1 and its analog have been used in diabetes treatment; however, the direct alteration of gene expression profile in human islets induced by GLP-1 has not been reported. In present study, transcriptional gene expression in the liraglutide-treated human islets was analyzed with 12 human U133A chips including 23000 probe sets. The data compared between liraglutide and control groups showed a significant difference on glucose-induced insulin secretion, rather than viability. Microarray analysis identified 7000 genes expressed in human islets. Eighty genes were found to be modulated by liraglutide treatment. Furthermore, the products of these genes are proteins involved in binding capability, enzyme activity, transporter function, signal transduction, cell proliferation, apoptosis, and cell differentiation. Our data provides a set of information in the complex events, following the activation of the GLP-1 receptor in the islets of Langerhans.

## 1. Introduction

GLP-1 is synthesized by the intestinal L-cells and it has been known to stimulate insulin release in the postprandial state, to inhibit glucagon release, and to slow down the rate of gastric emptying, along with acid secretion [1]. In addition to these well-known functions, novel properties of GLP-1 on maintaining beta-cell mass have been identified recently. In glucose-intolerant Wistar rats, GLP-1 reverses an age-dependent *β*-cell abnormalities, and this is associated with an expansion of *β*-cell mass via islet cell neogenesis [2]. Similarly, Exendin-4, a GLP-1 receptor agonist, stimulates both beta-cell replication and neogenesis, resulting in increased beta-cell mass and improved glucose tolerance in diabetic rat created by partial subpancreatectomy [3]. A GLP-1-dependent differentiation of pancreatic precursor cells into mature *β*-cells has also been proposed [4, 5]. Finally an inhibitory effect on islet cell apoptosis has been observed in the pancreas of animal models of diabetes, as well as in *β*-cell lines and isolated human islets in culture [6–8]. All these data suggested that treatment of human islets with exogenous GLP-1 could improve function and survival of pancreatic islets. Indeed, it has been demonstrated by several groups that GLP-1 enhances *β*-cell mass by both promoting islet cell neogenesis and inhibiting beta-cell apoptosis [8, 13]. However, the elucidation of the molecular and cellular mechanisms regulating this complex set of diverse biological actions is still very marginally understood.

To evaluate GLP-1 efficacy on human islets and to identify GLP-1 induced gene profile as well as the related signal pathways, we cultured human islets isolated from cadaveric donors with liraglutide [9], a long-lasting GLP-1 analog and investigated the gene expression profile after the exposure of human islets to GLP-1 by microarray analysis. We demonstrated that in response to activation of the GLP-1 receptor, a coordinated expression of factors regulating biological process took place. This paper provides a preliminary map on the intracellular responses of islet cells to GLP-1.

## 2. Materials and Methods 

### 2.1. Materials

Liberase-HI purified enzyme blend was purchased from Boehringer Mannheim (Indianapolis, IN); dithizone was purchased from Sigma (St. Louis, MO). Human U133A C hips were obtained from Affymetrix, INC (Santa Clara, CA). Medium and RNA extraction kit were purchased from Qiagen (QIAGEN Inc., Valencia, C A). Live/Dead Viability/Cytotoxicity Kit and streptavidin-phycoerythrin were from Molecular Probe (Eugene, OR). GLP-1 was purchased from American Peptide Co. (Sunnyvale, CA). Ficoll Cobe 2991 was purchased from COBE BCT, Inc. (Lakewood, CO). Tissue culture plastic ware was obtained from Corning Coaster Corporation (Cambridge, MA). Fetal calf serum and SuperScript Choice system were from Life Technologies, Inc. BRL (Rockville, MD). M199 medium was obtained from Gibco-BRL (Gaithersburg, MD). Bradford reagents for protein detection were obtained from Bio-Rad (Richmond, CA). Oligo-(dT)_24_ anchored T7 primer was obtained from Amersham Pharmacia Biotech (Piscataway, NJ). Phase Lock Gels were purchased from 5-prime-3-prime, Inc. (Boulder, CO). The BioArray high yield RNA transcript labeling kit was purchased from Enzo Diagnostics, Inc. (Farmingdale, NY). Biotinylated antistreptavidin was purchased from Vector Laboratories, Inc. (Burlingame, CA). PCR reagents, the software for the design of primers and TaqMan probes (Express V1.5 software), and the ABI PRISM 7700 Sequence Detector were from Applied Biosystems Inc. (Foster City, CA).

### 2.2. Islet Isolation, Purification, and Quantification

Human pancreases were recovered from cadaveric donors. Pancreas were placed in cold UW (University of Wisconsin) solution on ice and immediately transported to the islet isolation laboratory for processing and selected with criteria of having at least 10 h of cold storage in UW. All organs were processed using identical isolation techniques of collagenase digestion and Ficoll purification. Briefly, human pancreases were placed in a customized perfusion apparatus and injected with liberase-HI. They were then transferred to a continuous digestion device for mechanical disrupt and enzymatic digestion until the majority of the islets were free from exocrine tissue. Samples of pancreatic tissue were evaluated for purity during the digestion after staining with dithizone. Pancreatic digest containing endocrine and exocrine tissue was purified by continuous gradients centrifugation of Ficoll Cobe 2991. Islet-enriched layers were then selected and collected. Islet recovery after purification was assessed in duplication by counts of dithizone-stained aliquots of the final suspension of tissue. The purity of the preparations was assessed by comparing the relative quantity of dithizone-stained endocrine tissue divided by the unstained tissue. The viability of isolated cells was 87 ± 6%, and their purity was ≥57 ± 4%.

### 2.3. Living/Dead Cell Determination

Cell viability was determined using a Living/Dead Viability/Cytotoxicity Kit. Briefly, isolated islets were washed twice with P BS and 50 *μ*L aliquots were placed in the 96-well plate. Then 10 *μ*L of solution component A (Calcein AM) and component B (Ethidium homodimer-1) in Live/Dead Viability/Cytotoxicity K it were added to each well. After 30 min, islets were examined under a microscope. Living cells were identified by a green staining (Calcein AM staining), while dead cells showed a brown nuclear staining (ethidium homodimer-1 staining).

### 2.4. Incubation of Isolated Islets with GLP-1

Isolated islets were cultured, in 75-mL flasks, in the presence of M199 medium with 100 *μ*g/mL penicillin, 50 *μ*g/mL streptomycin, and 10% of fetal calf serum at 37°C under humidified conditions with 5% CO_2_. After 4 h, the islets were washed twice with M199 without FBS and antibiotics. They were then treated with 10 nM of GLP-1 in M199 medium containing 16 mM glucose (without F BS) for 22 h. Islet pellets were collected by centrifugation at 1000 rpm for 3 min. RNA was isolated by using the Qiagen RN easy Mini kit and immediately stored at −80°C.

### 2.5. Detection of Insulin Secretion

After 22 h of culturing in the presence of GLP-1, or vehicle, insulin released into the medium was measured by RIA (radioimmunoassay). Total cellular protein content was measured using the method of Bradford Assay. The amount of proteins measured served as a correction factor for determining the relative amount of insulin released into the culture medium by each individual culture condition.

### 2.6. Microarray Analysis

Affymetrix arrays (Human U133A) were used for mRNA expression profiling. Experimental procedures for gene chips were performed according to the Affymetrix Gene Chip Expression Analysis Technical Manual. Briefly: double-stranded cDNAs were synthesized using the SuperScript Choice system and an oligo-(dT)_24_ anchored T7 primer. Two samples (duplicate) of 5 *μ*g of total RNA from each sample were used to start the synthesis of cDNA. Double-stranded cDNA products were purified by phenol:chloroform:isoamyl alcohol (25 : 24 : 1 saturated with 10 mM Tris-HC l, pH8.0/1 mM EDTA), followed by extraction, phase separation with Phase Lock Gels, and ethanol precipitation. Biotinylated RNA was synthesized using the BioArray high yield RNA transcript labeling kit. Biotinylated RNA products were purified using Qiagen RNeasy columns and fragmented to a size of 30 to 200 nucleotides. A total of 15 ug biotinylated fragmented RNA was then hybridized with Affymetrix GeneChip arrays (Human, U133A). After washing, the arrays were stained with streptavidin-phycoerythrin, signal amplified by biotinylated antistreptavidin, and then scanned on an Agilent Gene array scanner. The intensity for each signal of the array was captured with the Affymetrix GeneChip Software (MAS 5.0), according to standard Affymetrix procedures. The mRNA abundance was determined based on the average of the differences between perfect match and intentional mismatch intensities for each probe family. Gene induction or downregulation was evaluated for statistical significance using the software provided by Silicon Genetics' GeneSpring 5.0, Affymetrix DMT 3.0.

### 2.7. Data Conversion and Statistics

All array assays were used at least in duplicate and the results represented an average of duplicates to minimize the assay variation. Expression data obtained from image files by Affymetrix Microarray Suite 5.0 were scaled to 200 expression units as the median. Raw expression values were normalized within each chip by dividing the median expression value of each individual chip. For each gene, the expression values of the treated samples were further normalized across chips by dividing the mean expression values of the control samples. Statistical analysis was performed with MATLAB software and data was transformed by natural log format. Differentially expressed genes were selected by three methods: (1) *t*-test (*P* < 0.05); (2) a density score greater than 100 for positively identified genes; (3) more than twofold differences from control. All identified genes were annotated based on NetAffy database and functionally classified by gene ontology.

### 2.8. Real-Time PCR

The expression of selected genes identified by Affymetrix GeneChip analysis was further verified by real-time PCR (with TaqMan technology) on an ABI Prism 7700 Sequence Detection System. PCR primers and TaqMan probes were designed with Primer Express V1.5 software, based on gene sequences downloaded from the GenBank or NetAffx web sites. PCR primers used for the real time PCR are listed in Table 1. TaqMan probes were labeled with 6-carboxy-fluorecein (6-FAM) as the reporter dye and 6-carboxy-tetramethyl-rhodamine (TAMR) as the quencher dye. Real-time PCR was performed in a two-step process. In the first step, sample RNA (0.1 *μ*g) or reference RNA was reverse transcribed in a volume of 100 *μ*L containing TaqMan RT buffer, 5.5 mM MgCl_2_, 500 *μ*M of each dNTP, 2.5 *μ*M random hexamers, 0.4 U/*μ*L RNase inhibitor, and 1.25 U/*μ*L MultiScribe Reverse Tanscripatase at 25°C for 10 min, 48°C for 30 min, and 95°C for 5 min. In the second step, real-time PCR was carried out in a MicroAmp Optical 96-well plate using TaqMan Gold PCR reagents. Each well contained 5 *μ*L of reverse-transcribed cDNA, TaqMan buffer A, 5.5 mM MgCl_2_, 200 *μ*M each of dATP/dC TP/dGTP, 400 *μ*M dUTP, 900 nM each of forward and reverse primers, 250 nM TaqMan probe, 0.01 U/*μ*L AmpErase UNG, and 0.025 U/*μ*L AmpliTaq Gold DNA polymerase in a total volume of 50 *μ*L. The thermal cycling conditions for real-time PCR were: a) 50°C for 2 min, b) 95°C for 10 min, and c) 40 cycles of melting (95°C, 15 sec) and annealing/extension (60°C, 60 sec). PCR reactions were monitored in real time using the ABI P RISM 7700 Sequence Detector. A standard curve for each target gene was generated with reference RNA. Relative quantification of gene expression was determined using the standard curve method as described in ABI's User Bulleting #2.

## 3. Results

### 3.1. Islet Viability and Responsiveness to GLP-1

**Figure 1:**
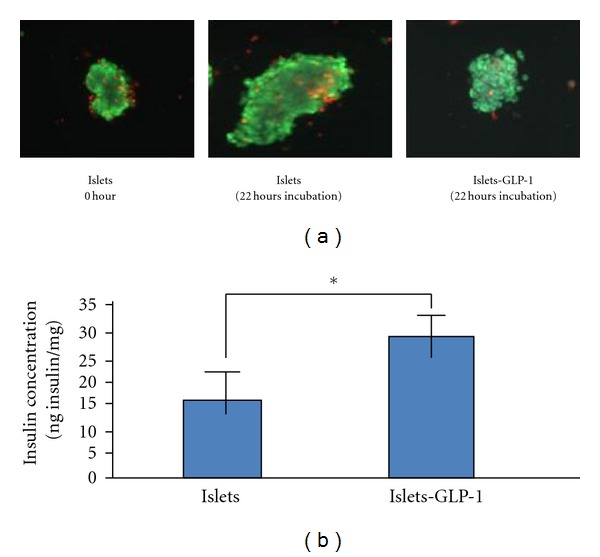
Viability and function of isolated human islets. Human islets were cultured in medium containing GLP-1 (10 nM), or vehicle for 22 h. (a) Cell viability was determined using the Living/Dead Viability/Cytotoxicity Kit. Living cells were identified by a green staining (Calcein AM staining), while dead cells were identified by a brown nuclear staining (Ethidium homodimer-1 staining). (b) The insulin accumulation into the culture medium was determined by RIA, and normalized to the total protein content in the cell pellet. Statistical significance of the data was evaluated by Student's *t-*test. *= *P* < 0.01.

Islets viability was determined by the Living/Dead Viability/Cytotoxicity assay. Living cells were identified by a green staining (Calcein AM staining), while a brown nuclear staining distinguished the dead cells (Ethidium homodimer-1 staining). We observed a majority (>90%) of islet cells viable, with no significant difference in the proportion of live versus dead cells between islets cultured in the presence of GLP-1, and control groups (Figure 1(a)).

The concentration of insulin in the medium, measured by RIA, and its abundance, normalized for by the total concentration of proteins in each individual culture are listed in Figure 1(b). There was 28.87 ± 3.29 ng insulin/mg of protein in the culture medium of islets grown in the presence of GLP-1 versus a 15.43 ± 3.6 ng insulin/mg of protein in the culture medium of islets grown in the control culture (no GLP-1). Statistic analysis indicates the difference between two status is significant (*P* < 0.01).

### 3.2. Genes Regulated by GLP-1

A total of 24 microarrays were employed to evaluate the gene expression profile associated with the exposure of human islets to GLP-1. After 22 h induction in a medium containing 10 nM GLP-1, more than 7000 gene transcripts were detected. Among them, the 80 genes (1%) with differential expression by 2-fold or more (up- or downregulation) are listed in Tables 1 and 2. The top 5 genes found upregulated by GLP-1 are: S EMA3C (sema domain, immunoglobulin domain (Ig), short basic domain, secreted, (semaphorin) 3C), 3.172-folds; RBBP6 (retinoblastoma binding protein 6], 3.147-folds; BARX1 (BarH-like homeobox 1), 3.122-folds; S100A9 (S100 calcium binding protein A9 (calgranulin B)), 2.940-folds; and DNAM-1 (adhesion glycoprotein), 2.935-folds, respectively. The top 5 genes found down-regulated by GLP-1 are: KCNJ15 (potassium inwardly-rectifying channel, subfamily J, member 15), 0.306-folds; UTS2 (urotensin 2), 0.299-folds; S LC7A6 (solute carrier family 7 (cationic amino acid transporter, y+ system), member 6), 0.260-folds; HSA9947 (putative ATPase), 0.231-folds; and ARHGEF9 (Cdc42 guanine nucleotide exchange factor (GEF) 9), 0.169-folds.

### 3.3. Validation of Microarray Data by Real-Time PCR

**Figure 2:**
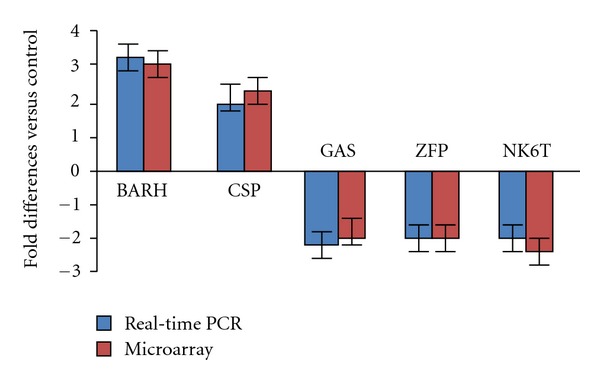
Results comparison between microarray and real-time-PCR. Five selected transcripts among those identified by microarray analysis were validated by the real-time-PCR analysis. The three transcripts selected were: BarH-like homeobox 1 (BARH); chondroitin sulfate proteoglycan (CSP); growth arrest-specific 2 (GAS); zinc finger protein 185 (LIM domain) (ZFP); NK6 transcription factor (NK6T). The graph shows the folds of induction or suppression as derived by the two methods. The data are expressed as the means ± SE of three independent analyses.

The alteration of gene expression by GLP-1 induction, as observed by microarray analysis, was further validated by real-time PCR. RNA samples from the same islet preparations were split and used for both microarray analysis and real-time PCR. We verified mRNA expression of 5 genes, two upregulated genes (BarH-like homeobox 1 and chondroitin sulfate proteoglycan) and three downregulated genes (growth arrest-specific 2, zinc finger protein 185, and NK6 transcription factor), responding GLP-1 induction. We were able to confirm the consistent results from real-time PCR and from microarray analysis (Figure 2).

### 3.4. Clustering of Genes Regulated by GLP-1

**Figure 3:**
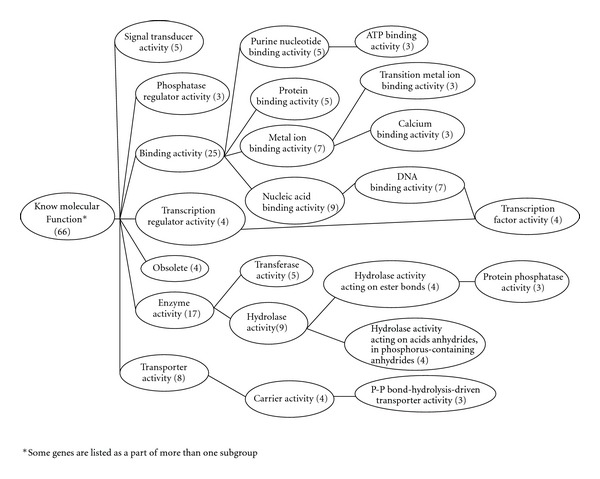
Functional clustering of islet genes regulated by GLP-1.

**Figure 4:**
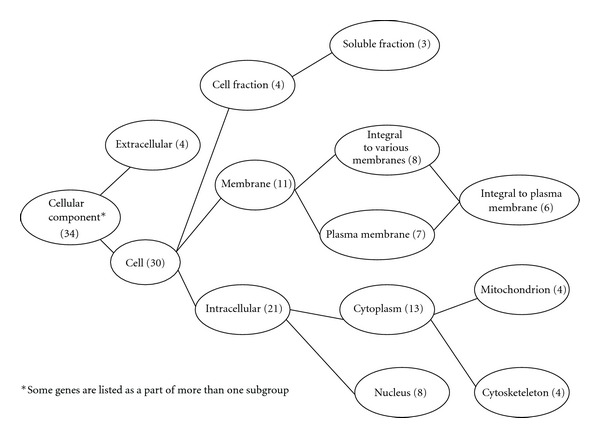
Cellular distribution of genes regulated by GLP-1.

**Figure 5:**
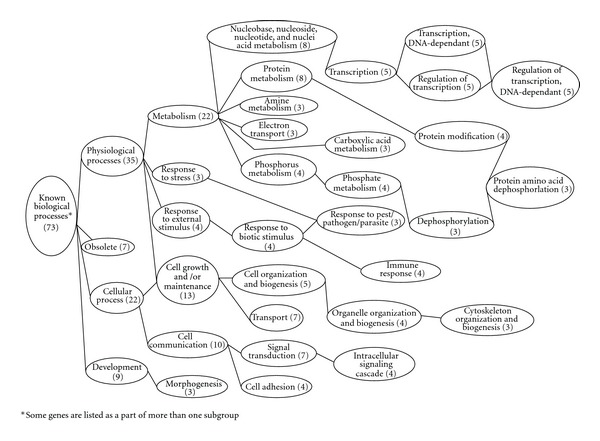
Biological function of genes regulated by GLP-1.

Among the 80 genes regulated by GLP-1, 66 were with known molecular functions, 34 were linked to cellular structural components; 73 were involved in various biological processes. Further analysis demonstrated that genes encoding for proteins, with protein-DNA or protein-protein binding capacity (*n* = 25) and enzyme activity (*n* = 17) were in two main subgroups (Figure 3). Genes encoding proteins with intracellular structure (*n* = 30) represented a main class for cellular component synthesis (Figure 4). Genes involved in physiological process (*n* = 35), including metabolism, response to stress, and response to external stimuli and the genes involved in cellular processes (*n* = 22), as cell growth and cell-cell interaction, represented the genes with known biological processes (Figure 5). Functional analysis showed that GLP-1 widely modulated the expression of genes linked with cell adhesion/extracellular matrix, cell cycle, cytoskeleton/structural, enzymatic activity and metabolism, growth factors/hormones/cytokines, nucleotide processing, protein processing, receptors, signal transduction, transcription factors, and transporter proteins.

## 4. Discussion

We report the identification of a large set of genes whose expression is regulated by the binding of GLP-1 to its receptor localized on human islets. The elucidation of this complex gene-regulatory network is essential for a better understanding of the physiological and pharmacological effects of GLP-1, as well as for deciphering the mechanism(s) by which this peptide has such a diverse repertoire of actions on islet cells. The effect of GLP-1 on islet cells has been investigated with rodent cell lines, as well as murine and human islets [10, 11]. From these researches, most data suggested that GLP-1 promoted beta-cell mass by increasing beta-cell proliferation, inhibiting beta-cell apoptosis, and inducing the differentiation of beta-cell from progenitor cells. While the *in vivo *application of GLP-1 on the diabetic and aging animals suggested that multifactors might be involved and sounded complex on the mechanism, the *in vitro *treatment of GLP-1 on the isolated islets provided more direct evidence of GLP-1 effect on islets. Farrilaetc demonstrated that GLP-1 added to freshly isolated human islets preserved the cell morphology and function and was able to inhibit cell apoptosis [12]. This effect is associated with a higher expression of Bcl-2 and a lower expression of active Caspase 3. Similarly, Mancosuetc found that high glucose concentration and glucagon-like peptide 1 (GLP-1) were associated with the maintenance of either the insulin secretary patterns from the incubated monolayer cells, or the transcriptional marker expression associated with beta-cell like phenotypes [13]. Upregulated expression of PDX-1, PAX4, Glut-2, and GK was also detected in their long cultured islets, in the presence of GLP-1.

In this study, we observed that the expression of most proliferation-related genes was associated with GLP-1 induction. These GLP-1 upregulated, proliferation-related genes include: the prolactin receptor, which has been proposed to regulate islet mass expansion during pregnancy [14]; retinoblastoma binding protein-6 (RBBP6) is a multifunctional protein found ostensibly in all eukaryotes but not in bacteria, which is implicated in a diverse set of cellular functions including mRNA metabolism, regulation of the cell cycle, tumour igenesis and development. [15]; CSPG2, which regulates cell motility and growth under P53 activation [16]; S100A4 can act as a novel cardiac growth and survival factor and may have regenerative effects in injured myocardium [17]; CYLD, which acts as tumor suppressor gene [18]; SEMA3a, which inhibits the binding of the VEGF with 165-amino acids (VEGF-165) to np1 and was reported to inhibit angiogenesis [19]. We were unable to observe the significant alteration of bcl-2, P ik3, and Irs2 reported by others groups with cell line and/or animal models [20, 21].

Antiapoptosis is another important biological event modulated by GLP-1 [22]. Our microarray analyses observed 3 apoptosis-related genes (CYLD, RBBP6, and PNUTL2) upregulated, and one (GAS2) downregulated. CYLD (the familial cylindromatosis tumor suppressor gene) enhances the activation of the transcription factor NFkapa-B [23], RBBP6, (which binds to the retinoblastoma gene product pRB) [24], and PNUTL2 (which is an apoptosis-related protein in the TGF-beta signaling pathway) [25]. GAS2 plays an important role in apoptosis by acting as a cell-death gene substrate for caspases [26]. These data suggest a conflict function of GLP-1 on apoptosis regulation. Increasing CYLD promotes apoptosis and increasing PNUTL enhances cell death via TGF. On the other hand, increased expression of RBBP6 and suppressed expression of GAS2 revealed an antiapoptosis effect of GLP-1.

These observations contradictory on apoptosis and differentiation might indicate complicated, but balanced functions between gene groups (pro- and antiapoptosis), rather than a simple and one direction to lead cell-death. They also indicate that cellular function regulation, such as apoptosis, differentiation, and so forth. are more complicated than what we speculated previously. To understand such a complicated regulation and balance, more advanced methodologies, such as gene expression profiling, may provide unique capacities or advantages to overview the cellular response to specific stimuli. Most of islet differentiation factors are transcriptional factors, and several transcripts of these factors in GLP-1 treated islets have be identified in this study. GLP-1 increased the expression of BARX1, PAX4, and decreased the expression of MYB, NKx6-1, FCMD, and ZNF185. The homoebox gene Barx1 is highly expressed in prospective stomach mesenchyme and is required to specify this organ [27], while PAX4 plays a role in the differentiation and development of pancreatic islet cells [28]. This is consistent with Brun's observation of that Pax4 expresses in human pancreatic islets and is activated during mitoss and by GLP-1 treatment [29]. MYB is essential for mammary tumorigenesis and its upregulation is associated with estrogen receptor (ER)-positive breast cancer [30]; NKx6-1 is a popular transcription factor involved in differentiation and development of pancreatic islet *β*-cells [31]. Significantly elevated expression of INSM1 was reported to be associated with both the AR42J cell line and the primary cultured mouse acinar cells differentiation into insulin-positive cells [32]. However in our observations, the expression of NKx6-1 was downregulated in GLP-1 treated islets. Similarly, the reduced expression of FCMD, which was reported to be involved in brain development [33], and ZNF185, a tumor-suppressor protein associated with actin-cytoskeleton [34] and reported in prostate cancer, was observed in our studies. Beyond our expectation, we were unable to detect the expression of PDX-1, although it is among the most extensively studied pancreatic transcriptional factor [35], responding to GLP-1 induction.

This study provides, the first time, the gene expression profile of human islets regulated by GLP-1 induction. The data presented here further supports the complex and diversity effects of GLP-1 in the regulation of protein governing, beta-cell mass control, and metabolism. The elucidation of the signal pathway triggered by GLP-1 may provide a scientific basis for molecular target identification, new drugs design, and diabetes disorder treatment.

## Figures and Tables

**Table 1:** Primers sequences used for RT-PCR.

Genes	Primers sequence (5′-3′)	U133A accession number and primer location on cDNA
BarH-like homeobox 1		219845_at
	TGGGC TCTAACC TGGGAGACT	21F
	GAGCTCAGGGTAGAGACTGTAGCTTC	83R
Chondroitin sulfate proteoglycan 2 (versican)		211571_s_at
	TTTAAAAATTCCTCATCAGCAAAGG	67F
	TCATGTTTGGATGTATTTATTGAATTGTC	19R
Growth arrest-specific 2		205848_at
	TGC TATGCTTTCAAGTAAAGTAAATTCAC	64F
	CAGCCCTGTCCCAGGTATAC AA	148R
Zinc finger protein 185 (LIM domain)		203585_at
	CGTTGGTGAGAAGGTGCCTATC	11F
	TCCACTGGTCCTGCACTGAG	62R
NK6 transcription factor related, locus 1 (Drosophila)		221366_at
	AGAAGCAGGACTCGGAGACAGA	1F
	TGCTGGACTTGTGCTTCTTCAA	138R

**Table:** (a)

Symbol	Name	Chromosome location	FC	*P* (*t*-test)
	Homo sapiens cDNA FLJ39067 fis, clone NT2RP7014910.		3.240	0.027
SEM A3C	Sema domain, immunoglobulin domain (Ig), short basic domain, secreted, (semaphorin) 3C	7q21-q31	3.172	0.031
RBBP6	Retinoblastoma binding protein 6	16p 12-p11.2	3.147	0.032
BARX1	BarH-like homeobox 1	9q12	3.122	0.020
S100A9	S100 calcium binding protein A9 (calgranulin B)	1q21	2.940	0.006
DNAM-1	Adhesion glycoprotein	18q22.3	2.935	0.007
	Homo sapiens cDNA FLJ14046 fis, clone HEMBA1006461.		2.907	0.006
	Homo sapiens cDNA FLJ10906 fis, clone OVARC1000035.		2.829	0.016
SEC15B	Sec15B protein	2p12	2.776	0.026
HIST1H3I	Histone 1, H3i	6p22-p21.3	2.773	0.001
PRLR	Prolactin receptor	5p14-p13	2.698	0.039
CYLD	Cylindromatosis (turban tumor syndrome)	16q11.2	2.672	0.031
SULT1C1	Sulfotransferase family, cytosolic, 1C, member 1	2q11.1-q11.2	2.586	0.034
	Homo sapiens, clone IMAGE: 4702418, mRNA		2.522	0.016
PTPN7	Protein tyrosine phosphatase, non-receptor type 7	1q32.1	2.504	0.017
CCL3	Chemokine (C–C motif) ligand 3	17q11-q21	2.500	0.000
S100A4	S100 calcium binding protein A4 (calcium protein, calvasculin, metastasin, murine placental homolog)	1q21	2.398	0.026
	Homo sapiens, clone IMAGE: 4471726, mRNA		2.364	0.020
KIAA1076	KIAA1076 protein	12q24.31	2.310	0.020
APOBEC3G	Apolipoprotein B mRNA editing enzyme, catalytic polypeptide-like 3G	22q13.1-q13.2	2.301	0.043
TRPM3	Transient receptor potential cation channel, subfamily M, member 3	9q21.11	2.265	0.027
	Homo sapiens mRNA; cDNA DKFZp434E2423 (from clone DKFZp434E2423)		2.225	0.040
PNUTL2	Peanut-like 2 (Drosophila)	17q22-q23	2.201	0.020
CSPG2	Chondroitin sulfate proteoglycan 2 (versican)	5q14.3	2.198	0.037
COL14A1	Collagen, type XIV, alpha 1 (undulin)	8q23	2.189	0.025
MGC27165	Hypothetical protein MGC27165	14	2.111	0.021
C16orf5	Chromosome 16 open reading frame 5	16p13.3	2.106	0.016
FLJ23342	Hypothetical protein FLJ23342	11q24.2	2.089	0.024
SBBI31	SBBI31 protein	5q23.2	2.085	0.029
PAX4	Paired box gene 4	7q32	2.082	0.040
FLJ10140	Hypothetical protein FLJ10140	22q13	2.077	0.029
KIAA0999	KIAA0999 protein	11q23.3	2.048	0.015
	Homo sapiens mRNA full length insert cDNA clone EUROIMAGE 362780.		2.008	0.036

**Table:** (b)

Symbol	Name	Chromosome location	FC	*P* (*t*- test)
GAS2	Growth arrest-specific 2	11p14.3-15.2	0.498	0.045
SUOX	Sulfite oxidase	12q13.13	0.496	0.034
KIAA0469	KIAA0469 gene product	1p36.23	0.496	0.026
DNAJB6	DnaJ (Hsp40) homolog, subfamily B, member 6	7q36.3	0.495	0.022
RBM 9	RNA binding motif protein 9	22q13.1	0.490	0.034
MYB	v-myb myeloblastosis viral oncogene homolog (avian)	6q22-q23	0.488	0.030
CLASP2	Cytoplasmic linker associated protein 2	3p 22.2	0.487	0.047
MGC5297	Hypothetical protein M GC5297	5p15.3-p15.2	0.486	0.039
PPP2R4	Protein phosphatase 2A, regulatory subunit B′ (PR 53)	9q34	0.484	0.031
PIP5K1C	Phosphatidylinositol-4-phosphate 5-kinase, type I, gamma	19p13.3	0.482	0.022
ZFHX1B	Zinc finger homeobox 1b	2q22	0.479	0.028
ADCY9	Adenylate cyclase 9	16p13.3	0.463	0.043
FLJ12788	Hypothetical protein FLJ12788	2p12	0.458	0.015
TM 6SF1	Trans membrane 6 superfamily member 1	15q24-q26	0.447	0.043
TBCE	Tubulin-specific chaperone e	1q42.3	0.446	0.035
GTF2H2	General transcription factor IIH, polypeptide 2, 44k Da	5q12.2-q13.3	0.438	0.007
PRO1331	Hypothetical protein PRO1331	5q33.3	0.437	0.024
ZNF185	Zinc finger protein 185 (LIM domain)	Xq28	0.428	0.021
KIAA0460	KIAA0460 protein	1q21.2	0.427	0.021
DKFZP434B168	DKFZP434B168 protein	1p36.13-q42.3	0.426	0.042
FCMD	Fukuyama type congenital muscular dystrophy (fukutin)	9q31-q33	0.415	0.045
ABCF2	ATP-binding cassette, subfamily F (GCN20), member 2	7q36	0.414	0.012
LM O7	LIM domain only 7	13q21.33	0.413	0.039
SULF1	Sulfatase 1	8q13.1	0.412	0.033
KIAA0090	KIAA0090 protein	1p36.13	0.411	0.003
POR	P450 (cytochrome) oxidoreductase	7q11.2	0.407	0.015
SE70-2	Cutaneous T-cell lymphoma tumor antigen se70-2	13q22.1	0.406	0.030
NKX6-1	NK6 transcription factor related, locus 1 (Drosophila)	4q21.2-q22	0.403	0.006
LOC51231	VRK3 for vaccinia related kinase 3	19q13	0.403	0.002
BYSL	Bystin-like	6p21.1	0.401	0.032
HIP1R	Huntingtin interacting protein-1-related	12q24	0.396	0.012
PPP2R4	Protein phosphatase 2A, regulatory subunit B′ (PR 53)	9q34	0.387	0.046
WASL	Wiskott-Aldrich syndrome-like	7q31.3	0.383	0.032
TIMM 17A	Translocase of inner mitochondrial membrane 17 homolog A (yeast)	1q32.1	0.378	0.032
ARL4	ADP-ribosylation factor-like 4	7p21-p15.3	0.375	0.002
LOC51204	Clone HQ0477 PRO0477p	17q24.1	0.363	0.023
KIAA1023	KIAA1023 protein	7p22.3	0.357	0.017
CPT1A	carnitine palmitoyl transferase 1A (liver)	11q13.1-q13.2	0.351	0.021
RAD51C	RAD51 homolog C (S. cerevisiae)	17q22-q23	0.347	0.022
ITGB3	integrin, beta 3 (platelet glycoprotein IIIa, antigen CD61)	17q21.32	0.345	0.008
MGC4309	hypothetical protein M GC4309	1q32.1	0.336	0.007
	ESTs, weakly similar to neuronal thread protein (Homo sapiens)		0.334	0.030
KCNJ15	potassium inwardly-rectifying channel, subfamily J, member 15	21q22.2	0.306	0.006
UTS2	urotensin 2	1p36	0.299	0.032
SLC7A6	solute carrier family 7 (cationic amino acid transporter, y+ system), member 6	16q22.1	0.260	0.016
HSA9947	putative ATPase	1p36	0.231	0.050
ARHGEF9	Cdc42 guanine nucleotide exchange factor (GEF) 9	Xq11.1	0.169	0.019

## References

[1] Doyle ME, Egan JM (2007). Mechanisms of action of glucagon-like peptide 1 in the pancreas. *Pharmacology and Therapeutics*.

[2] Gaddy DF, Riedel MJ, Pejawar-Gaddy S, Kieffer TJ, Robbins PD (2010). In vivo expression of HGF/NK1 and GLP-1 from dsAAV vectors enhances pancreatic *β*-cell proliferation and improves pathology in the db/db mouse model of diabetes. *Diabetes*.

[3] Xu G, Stoffers DA, Habener JF, Bonner-Weir S (1999). Exendin-4 stimulates both *β*-cell replication and neogenesis, resulting in increased *β*-cell mass and improved glucose tolerance in diabetic rats. *Diabetes*.

[4] Hui H, Wright C, Perfetti R (2001). Glucagon-like peptide 1 induces differentiation of islet duodenal homeobox-1-positive pancreatic ductal cells into insulin-secreting cells. *Diabetes*.

[5] Hui H, Tang YG, Zhu L (2010). Glucagon like peptide-1-directed human embryonic stem cells differentiation into insulin-producing cells Via Hedgehog, cAMP, and PI3K pathways. *Pancreas*.

[6] Boutant M, Ramos OH, Tourrel-Cuzin C (2012). Vasseur-cognet M.COUP-TFII controls mouse pancreatic *β*-cell mass through GLP-1-*β*-catenin signaling pathways. *PLoS One*.

[7] Hui H, Nourparvar A, Zhao X, Perfetti R (2003). Glucagon-like peptide-1 inhibits apoptosis of insulin-secreting cells via a cyclic 5′-adenosine monophosphate-dependent protein kinase A- and a phosphatidylinositol 3-kinase-dependent pathway. *Endocrinology*.

[8] Farilla L, Hongxiang H, Bertolotto C (2002). Glucagon-like peptide-1 promotes islet cell growth and inhibits apoptosis in Zucker diabetic rats. *Endocrinology*.

[9] Perry CM (2011). Liraglutide: a review of its use in the management of type 2 diabetes mellitus. *Drugs*.

[10] Tian L, Gao J, Weng G (2011). Comparison of exendin-4 on beta-cell replication in mouse and human islet grafts. *Transplant International*.

[11] Favaro E, Granata R, Miceli I (2012). The ghrelin gene products and exendin-4 promote survival of human pancreatic islet endothelial cells in hyperglycaemic conditions, through phosphoinositide 3-kinase/Akt, extracellular signal-related kinase (ERK)1/2 and cAMP/protein kinase A (PKA) signalling pathways. *Diabetologia*.

[12] Farilla L, Bulotta A, Hirshberg B (2003). Glucagon-like peptide 1 inhibits cell apoptosis and improves glucose responsiveness of freshly isolated human islets. *Endocrinology*.

[13] Mancuso F, Basta G, Calvitti M (2006). Long-term cultured neonatal porcine islet cell monolayers: a potential tissue source for transplant in diabetes. *Xenotransplantation*.

[14] Cypryk K, Vilsbøll T, Nadel I, Smyczyńska J, Holst JJ, Lewiński A (2007). Normal secretion of the incretin hormones glucose-dependent insulinotropic polypeptide and glucagon-like peptide-1 during gestational diabetes mellitus. *Gynecological Endocrinology*.

[15] Kappo MA, Ab E, Hassem F (2012). Solution structure of RING finger-like domain of retinoblastoma-binding protein-6 (RBBP6) suggests it functions as a U-box. *The Journal of Biological Chemistry*.

[16] Rahmani M, Wong BW, Ang L (2006). Versican: signaling to transcriptional control pathways. *Canadian Journal of Physiology and Pharmacology*.

[17] Schneider M, Kostin S, Strøm CC (2007). S100A4 is upregulated in injured myocardium and promotes growth and survival of cardiac myocytes. *Cardiovascular Research*.

[18] Courtois G, Gilmore TD (2006). Mutations in the NF-*κ*B signaling pathway: implications for human disease. *Oncogene*.

[19] Guttmann-Raviv N, Shraga-Heled N, Varshavsky A, Guimaraes-Sternberg C, Kessler O, Neufeld G (2007). Semaphorin-3A and semaphorin-3F work together to repel endothelial cells and to inhibit their survival by induction of apoptosis. *The Journal of Biological Chemistry*.

[20] Kushner JA, Simpson L, Wartschow LM (2005). Phosphatase and tensin homolog regulation of islet growth and glucose homeostasis. *The Journal of Biological Chemistry*.

[21] Park S, Dong X, Fisher TL (2006). Exendin-4 uses Irs2 signaling to mediate pancreatic *β* cell growth and function. *The Journal of Biological Chemistry*.

[22] Urusova IA, Farilla L, Hui H, D’Amico E, Perfetti R (2004). GLP-1 inhibition of pancreatic islet cell apoptosis. *Trends in Endocrinology and Metabolism*.

[23] Wang L, Baiocchi RA, Pal S, Mosialos G, Caligiuri M, Sif S (2005). The BRG1- and hBRM-associated factor BAF57 induces apoptosis by stimulating expression of the cylindromatosis tumor suppressor gene. *Molecular and Cellular Biology*.

[24] Gao S, Scott RE (2003). Stable overexpression of specific segments of the P2P-R protein in human MCF-7 cells promotes camptothecin-induced apoptosis. *Journal of Cellular Physiology*.

[25] Larisch S, Yi Y, Lotan R (2000). A novel mitochondrial septin-like protein, ARTS, mediates apoptosis dependent on its P-loop motif. *Nature Cell Biology*.

[26] Benetti R, Sal GD, Monte M, Paroni G, Brancolini C, Schneider C (2001). The death substrate Gas2 binds m-calpain and increases susceptibility to p53-dependent apoptosis. *EMBO Journal*.

[27] Woo J, Miletich I, Kim BM, Sharpe PT, Shivdasani RA (2011). Barx1-Mediated inhibition of Wnt signaling in the mouse thoracic foregut controls Tracheo-Esophageal septation and epithelial differentiation. *PLoS ONE*.

[28] Greenwood AL, Li S, Jones K, Melton DA (2007). Notch signaling reveals developmental plasticity of Pax4+ pancreatic endocrine progenitors and shunts them to a duct fate. *Mechanisms of Development*.

[29] Brun T, He KHH, Lupi R (2008). The diabetes-linked transcription factor Pax4 is expressed in human pancreatic islets and is activated by mitogens and GLP-1. *Human Molecular Genetics*.

[30] Miao RY, Drabsch Y, Cross RS (2011). MYB is essential for mammary tumorigenesis. *Cancer Research*.

[31] Nelson SB, Schaffer AE, Sander M (2007). The transcription factors Nkx6.1 and Nkx6.2 possess equivalent activities in promoting beta-cell fate specification in Pdx1+ pancreatic progenitor cells. *Development*.

[32] Zhang T, Saunee NA, Breslin MB, Song K, Lan MS (2012). Functional role of an islet transcription factor, INSM1/IA-1, on pancreatic acinar cell trans-differentiation. *Journal of Cellular Physiology*.

[33] Ohtsuka-Tsurumi E, Saito Y, Yamamoto T, Voit T, Kobayashi M, Osawa M (2004). Co-localization of fukutin and *α*-dystroglycan in the mouse central nervous system. *Developmental Brain Research*.

[34] Zhang JS, Gong A, Young CYF (2007). ZNF185, an actin-cytoskeleton-associated growth inhibitory LIM protein in prostate cancer. *Oncogene*.

[35] Hui H, Perfetti R (2002). Pancreas duodenum homeobox-1 regulates pancreas development during embryogenesis and islet cell function in adulthood. *European Journal of Endocrinology*.

